# Hair-Based Biomonitoring of Phthalate Metabolites and Triclosan in Childhood: Links with Metabolic and Endocrine Disorders

**DOI:** 10.3390/jox16050158

**Published:** 2026-08-25

**Authors:** Michail Koukakis, Stella Baliou, Athanasios Alegakis, Elena Vakonaki, Dimitra Volakaki, Ilianna Maniadaki, Eleftheria Papadopoulou, Dimitris Mamoulakis, Marilia Lioudaki, Emmanouil Paraskakis, Ioannis Germanakis, Aristides Tsatsakis, Manolis N. Tzatzarakis

**Affiliations:** 1 Laboratory of Toxicology, Medical School, University of Crete, 70013 Heraklion, Greece; medp2011978@med.uoc.gr (M.K.); alegkaka@uoc.gr (A.A.); volakaki.demetra@gmail.com (D.V.);; 2 Department of Pediatrics, University Hospital Heraklion, School of Medicine, University of Crete, 70013 Heraklion, Greecemarilia_lioudaki@hotmail.com (M.L.);; 3 Human Development and Health Science Faculty, Universidad ECOTEC, Samborondon 092302, Ecuador; 4 Biomedical Science and Technology Park, Sechenov I.M. First State Medical University, 119991 Moscow, Russia

**Keywords:** endocrine-disrupting chemicals, phthalate metabolites, triclosan, hair, biomonitoring, children, obesity, type 1 diabetes mellitus, hypothalamic-pituitary-related diseases

## Abstract

Background–Aim: Children represent a particularly susceptible population to endocrine-disrupting chemical (EDC) exposure; however, biomonitoring data in this population remain limited. To the best of our knowledge, this is the first study using hair biomonitoring to simultaneously assess the concentrations of phthalate metabolites (PMs), triclosan (TCS) and triclocarban (TCC) in children with obesity (OB), type 1 diabetes mellitus (T1D), and hypothalamic–pituitary-related diseases (HPRD), through hair biomonitoring. Methods: Liquid chromatography–mass spectrometry (LC-MS) was used to measure TCS, TCC and PM levels in head hair samples from 212 children, categorized into control, T1D, HPRD and OB groups. PMs and TCS levels were compared across groups and evaluated in relation to health issues, cosmetics exposure, dietary habits, cardiometabolic, and somatometric parameters. Results: Our hair biomonitoring results showed that the most commonly detected EDCs were MEHP, MBP, MiBP, and TCS, with detection frequencies of samples 95.3%, 84.4%, 79.7%, and 60.8%, respectively. Children with T1D had statistically significantly higher mean MEHP concentrations than the control group. Children with HPRD exhibited higher PM concentrations, primarily driven by elevated mean MEHP and MBP levels compared with the control group. In contrast, mean MiBP levels were lower in the OB group than in the control group. Regarding TCS levels, no statistically significant differences were observed across the disease groups compared with controls. TCS was inversely associated with somatometric parameters in the OB group. Subgroup analyses by age, sex, residence area, and maternal education were conducted to assess statistically significant differences in concentrations of PM and TCS. Conclusions: Overall, our biomonitoring results indicate group-specific concentration patterns of PMs and TCS among children with T1D, HPRD and OB groups compared to the control group. In particular, PM levels were higher in children with T1D and HPRD and lower in those with OB groups compared to the control group. As a result, this is the first study to assess PM and TCS concentrations in children across clinical groups, using hair matrix.

## 1. Introduction

In the modern industrialized environment of developed countries, endocrine-disrupting chemicals (EDCs) are omnipresent [1]. Exposure to these substances occurs through various routes, including ingestion (food and water intake), inhalation (air and dust), respiratory tract, with skin absorption (via personal care and packaging products) being the main route of EDC penetration [2,3,4,5].

Children are a population that is highly susceptible to EDC exposure due to variations in pharmacokinetics, physiology, anatomy, behavior and nutrition [6]. From a physiological and anatomical perspective, children are more susceptible to these chemicals due to their immature detoxification systems, lower body weight and a higher relative growth rate compared to adults, and their increased minute ventilation and calorie consumption per body surface area [7]. From a molecular perspective, even low concentrations of EDCs can impair DNA repair capacity, immunosurveillance mechanisms, liver metabolism through effects on detoxification processes, the nervous system through impacts on the blood–brain barrier and normal metabolism— that might not yet be fully formed [8]. For example, the hepatic cytochrome P450 enzymes are less abundant in children, thereby reducing the metabolism of environmental chemicals less effectively [9,10].

According to the Developmental Origins of Health and Disease (DOHaD) paradigm, exposure to EDCs during early developmental stages may trigger persistent metabolic changes, increasing disease risk later in life [11]. In accordance with the environmental obesogens concept [12,13,14], EDCs can trigger coordinated responses across metabolic organs, including the brain, liver, pancreas, gastrointestinal tract, and adipose tissue [15]. During this critical developmental period of childhood, EDCs can induce metabolic dysfunction by mimicking, opposing, or altering the production, transport, and metabolism of natural hormones, which have much lower receptor-binding capacity than that of natural ligands [16,17], thereby disrupting hormonal signaling pathways [18]. From a molecular perspective, EDCs contribute to metabolic disturbance by affecting adipogenesis, interfering with neuroendocrine signals responsible for appetite and satiety, triggering a long-term low-grade inflammatory state, altering the gut microbiome, impairing the function of thermogenic adipose tissue and impairing the hypothalamic–pituitary–thyroid (HPT) signaling cascade [19,20,21,22,23]. These metabolic alterations become stabilized through epigenetic modifications [24,25,26], including DNA methylation and histone modifications [27,28,29].

In the field of EDCs, phthalate metabolites (PMs), triclosan (TCS) and triclocarban (TCC) undergo rapid metabolism and excretion with short half-lives [30,31,32]. PMs are diesters of 1,2-benzenedicaraboxylic acid (phthalic acid), commonly detected in PVC, personal care products, medical items, toys and food packaging which are the main manufacturing sectors [33,34,35,36]. PMs are frequently used in the production of plastic products to increase their softness and flexibility. [37]. Due to their extensive use, PMs are among the most commonly identified environmental chemicals in human biomonitoring analyses, massive production and constant exposure. In addition, PMs have been detected in a range of coatings, varnishes, and personal care items (such as lotions, fragrances, and cosmetics) [38]. TCS and TCC are compounds known for their antibacterial properties and can be detected in personal care products such as soaps, sanitizers, and disinfectants [39,40]. Several biomonitoring studies have shown TCS presence in children [41,42]. The widespread use of multiple consumer products may expose children to both PMs and TCS [43].

Several epidemiological studies and meta-analyses have provided insights into the positive correlation between children’s exposure to EDCs and the development of metabolic or hypothalamus–pituitary gland-related disorders (HPRD), as evidenced by urine biomonitoring [44,45,46,47,48,49], suggesting the negative impact of EDCs on human health. In particular, the hypothalamus–pituitary gland–thyroid (HPT), hypothalamus–pituitary gland–gonads (HPG), hypothalamus–pituitary gland–adrenal (HPA) and hypothalamus–pituitary–somatotropic (HPS) axes are the primary targets of EDCs, which exert their effects by regulating various signaling and transduction pathways [50,51]. The dynamic crosstalk between several hypothalamic–pituitary signaling cascades has been highlighted [52].

To the best of our knowledge, this is the first study to apply hair-based biomonitoring of PMs, TCS, and TCC in children with diagnosed endocrine and metabolic conditions, while also examining differences in exposure burden according to gender, age, dietary habits, and cosmetics use. Many animal studies have provided experimental evidence on these endocrine compounds. Specifically, prenatal dibutyl phthalate exposure in mice has been shown to promote obesity in offspring with concurrent effects on glucose and cholesterol metabolism [53], while TCS has been linked to gut microbiome disruption in rats, a proposed contributor to childhood obesity risk [54]. For thyroid function, DEHP has been shown in animal models to interfere with HPT-axis signaling, including thyroid-stimulating hormone (TSH) receptor expression, thyrotropin-releasing hormone (TRH) receptor, thyroid peroxidase (TPO), sodium iodide symporter function (NIS), and deiodinase activity [55] while TCS lowers circulating thyroxine (T4) in rat samples during critical developmental windows [56]. Although animal studies have suggested potential associations between these endocrine disruptors, data from human studies remain limited. These gaps in the experimental literature—particularly for obesity (OB), type 1 diabetes mellitus (T1D) and hypothalamus–pituitary-related disorders (HPRD)— further support the value of the present study as hypothesis-generating groundwork for future mechanistic and epidemiological research in humans.

## 2. Materials and Methods

### 2.1. Reagents

The monobutyl phthalate (MBP), mono-isobutyl phthalate (MiBP), bis(3,5,5-trimethylhexyl) phthalate (BTMHP), mono-n-octyl phthalate (MOP), monobenzyl phthalate (MBzP), monoethyl phthalate (MEP) were purchased at 98% purity from Toronto Research Chemicals (TRC Inc., North York, ON, Canada). The mono(2-ethylhexyl) phthalate (MEHP), monomethyl phthalate (MMP), monocyclohexyl phthalate (MCHP) as well as triclosan (TCS) (98%) and triclocarban (TCC) (98%) were purchased from Sigma-Aldrich (St Louis, MO, USA). Phenobarbital was used as internal standard and was obtained from Lipomed AG (Arlesheim, Switzerland). Methanol and acetonitrile (LC-MS grade) were purchased from Fisher Scientific International, Inc. (Loughborough, UK). Ultrapure water was produced by a Direct-Q 3UV water purification system (Darmstadt, Germany).

### 2.2. Study Population Characteristics

The study population comprised children aged 2–16 years who were recruited from the University General Hospital of Heraklion in Crete. In collaboration with the doctors of Pediatric Clinics, children who were being followed up in the pediatric OB clinic, pediatric endocrinology and pediatric diabetes clinics and had already been diagnosed with T1D, OB and HPRD were recruited in the study. The control group consisted of children with no chronic diseases and came either from the general population or were recruited by the above-mentioned clinics during a visit for a reason that was not related to a metabolic or endocrinological problem. Children under 2 years of age and over 17 were excluded from the study. All children and their parents were informed about the purpose and scope of the study and written informed consent for their participation was obtained prior to sample collection and questionnaire completion. Age-specific informational infographic files were provided to the children and the research purposes of the study were explained to them.

### 2.3. Hair Sampling and Questionnaires

Scalp hair samples (total length) were collected during 2021–2022 and stored in closed envelopes, in a dry and dark place at room temperature until the analysis. In total, 212 children participated in the study, 51 of whom comprised the control group, 51 the T1D group, 51 the OB group and 59 the HPRD group with stratified sampling according to their age (indicatively, 2–10 and 11–16 years old) and to the chronic health problem they faced. The entire population of children was resident on the island of Crete, Greece.

With respect to the questionnaires, they were completed by the children, their parents and the children’s treating physicians. The first questionnaire included information regarding: (a) parents’ socioeconomic data (occupation, place of residence, education, etc.), (b) children’s somatometric data (age, weight, height) (c) the frequency of use of cosmetics and care products, (d) the type and usage frequency of food storage in the house and the children’s dietary habits: weekly frequency consumption of meat, fish, dairy products, fruit, vegetables, sweets, pasta, rice, bread, canned foods, condiments, ready meals, juices, snacks, beverages, (e) preferable packaging of the previously mentioned products (plastic, metal, aluminum, etc.).

With regard to the second questionnaire, it was completed by the children’s treating physicians and it included questions regarding children’s somatometric data (weight, height, body mass index (BMI), waist circumference (WC), blood pressure (mm/HG)) and children’s medical history (type of disease and age of onset). This study was approved by the Research Ethics Committee of the University of Crete (2/15.01.2021) and the Scientific Council of University General Hospital of Heraklion (361/12-05-2021). 

### 2.4. Sample Preparation

#### PM, TCS and TCC Extraction from Hair

The analytical protocol used for the analysis of children’s hair samples has been developed and described elsewhere [57,58]. Hair samples were washed twice with 2 mL of water and 2 mL of methanol to remove any external contamination and then they were dried at 50 ± 5 °C. Subsequently, the hair was cut into a few mm, 100 mg were weighed and 25 ng of IS was added, and extraction of the analytes was carried out with 2 × 2 mL of methanol in ultrasonic bath at 50 ± 5 °C for 2 × 2 h. The organic phase (total 4 mL) was collected, evaporated to dryness under a nitrogen stream at 35 °C and reconstituted in 0.1 mL of methanol prior to analysis by liquid chromatography–atmospheric.

### 2.5. Instrumental Analysis

#### 2.5.1. Instrumentation of LC-MS

The analysis of TCS, TCC and PM was carried out in a Shimadzu LC-MS 2010EV (Kyoto, Japan) and the separation of analytes was achieved on a Supelco Discovery C18 column (25 cm × 4.6 mm, 5 μm) (Supelco, Bellefonte, PA, USA) at a flow rate 0.6 mL/min and the oven temperature was 30 °C. The duration of chromatographic analysis was 26 min. The mobile phase of PM, TCS and TCC consisted of 5 mM ammonium acetate (solvent A) and acetonitrile containing 0.1% formic acid (solvent B). Mass spectrometer was coupled with an APCI ion source and the monitoring was achieved in selected ion monitoring (SIM) in negative mode. Detector voltage was set at 1.5 kV, drying gas at 0.02 MPa, interface temperature was 400 °C, CDL temperature was 200 °C, nebulizing gas flow was set at 2.5 L/min and heat block temperature was 200 °C.The target ion (used for the quantitative determination of the analytes), the Q1 ion (employed for qualitative confirmation) and the retention times (Rt) are presented in Table 1. The software LCMS solution Ver3 (Shimadzu) was used during the analysis and quantification.

#### 2.5.2. Standard Solutions and Spiked Human Samples

Stock solutions of each compound were prepared at a concentration level of 100 ppm. Mixed working solutions of PM, TCS and TCC were prepared at concentrations 5, 1.0 and 0.1 ppm and stored at −20 °C.

Human hair with no detected levels of the aforementioned analytes or levels below LOQ was pooled and used as samples for blank hair. The pooled samples were spiked at concentrations 0, 10, 25, 50, 100, 250, 500 and 1000 pg/mg for PMs and 0, 25, 50, 100, 250, 500, 1000 and 2000 pg/mg for TCS and TCC. They were extracted as the authentic samples, analyzed and used for the preparation of the spiked calibration curves, as well as for the validation of the method.

### 2.6. Statistical Analysis

Data entry was performed using Microsoft Excel (2020) while the statistical analysis was performed via IBM SPSS Statistics 23.0. The levels of the EDCs of the study were expressed as means, standard deviation (SD) and 1st and 3rd quartiles. The frequency of positive samples (>LOD) was expressed as counts and percentages. Differences in frequency of discrete demographic variables were examined using Pearson’s chi-square test. Differences in levels of EDCs between groups were examined using non-parametric tests such Mann–Whitney for two group or Kruskal–Wallis for more than two groups. Effects on MEHP, MBP, MiBP and TCS levels from each disease group were estimated using multiple linear regression after adjustment with child’s age, residence and mother’s education. Correlation of continuous data (e.g., concentration vs. somatometrics) were expressed using Spearman’s rho. A Bonferroni correction was also applied in correlation pairs. All negative samples of the study (<LOD) were set as LOD/$\sqrt{2}$ for the measurement of concentrations when imputation of data was referred. The level of statistically valid hypotheses was set at *p* = 0.050.

### 2.7. Somatometric and Clinical Measurements

To adjust for physiological variations associated with sex, age and physical development among the study participants, raw growth and blood pressure measurements were converted to standardized SD scores (z-scores). Somatometric variables, including weight, height and BMI, were converted to z-scores using the 2000 Centers for Disease Control and Prevention (CDC) Growth Charts reference population [59]. Unlike percentiles, which are adversely affected by mathematical non-linearity and compression at extreme values, z-scores create a linear, ratio scale that increases statistical power and maintains the distribution parameters required for the parametric framework [1]. For the blood pressure values, raw systolic (SBP) and diastolic (DBP) values were baseline-corrected using a triple-standardization approach. Given the fact that pediatric blood pressure is structurally confounded by body size, a child’s height z-score (derived from the CDC reference) was first computed. Height z-score, along with age and biological sex, was then used to calculate precise SBP and DBP z-scores using the regression equations established by the American Academy of Pediatrics (AAP) Clinical Practice Guidelines [60].

### 2.8. Cosmetics Exposure Index (CEI)

To assess children’s consumer exposure, a cosmetics exposure index (CEI) was generated based on questionnaire items that included the use of personal care products, specifically hair cleaning lotions (e.g., shampoos and body washes), body deodorants (e.g., roll-on, spray), perfume and hair cosmetics. Each item was scored on a scale from 0 to 3 based on frequency of use, where 0 indicates no use, 1 indicates 1–3 times per week, 2 indicates 4–5 times per week, and 3 indicates more than 5 times per week. This index quantified the frequency and variety of cosmetic product usage among the study participants. The primary objective was to investigate the relationship between cosmetics exposure—as measured by the CEI—and EDC levels in children’s bodies. Statistical analyses were performed to evaluate the correlation between CEI scores and EDC concentrations. Statistical correlations between children’s CEI frequencies and EDC levels were estimated using Spearman rank correlation coefficient (r) and the corresponding significance level (*p*-value).

### 2.9. Dietary Exposure Index (DEI)

For dietary assessment of children, a dietary exposure index (DEI) was generated from a food frequency questionnaire (FFQ). The dietary components were sorted into pre-specified contamination profiles. Regarding the FFQ, children and their parents answered questions about their food consumption, such as legumes, fruits, vegetables, starchy vegetables (potato, sweet potato), pork, poultry, beef, fresh and frozen fish, milk, cheese, yoghurt, pasta, rice, bread, cereals, eggs, canned foods, butter, margarine, condiments, honey, marmalade, sweets, chocolate, juices, snacks, beverages, fast food and ready meals. The responders reported their weekly consumption frequency. Each question was scored on a scale of 0–3, where 0 indicates no use, 1 indicates 1–2 times per week, 2 indicates 3–4 times per week, and 3 indicates up to 5 times per week. The scores were assigned to reflect the respective intake frequencies.

We sorted these foods into the following eleven categories: dairy (milk, cheese, yogurt), meat (pork, poultry, beef), fish (fresh and frozen), starchy foods (pasta, rice, bread, potato, sweet potato), processed food (snacks, cereals, fast food, ready meals, canned foods), fruits/veg (fruits, vegetables, marmalade, honey), legumes, fat and condiments (TFC) (butter, margarine, condiments), sweets (sweets, chocolate), refreshments (bottled juices, beverages) and eggs. A composite index was derived to operationalize the total consumption of the eleven categories. This index quantified the frequency and variety of dietary product usage among the study children. The primary objective was to investigate the relationship between dietary exposure—as measured by the DEI—and PM levels in children’s bodies.

Statistical correlations between children’s dietary frequencies and PM levels were estimated using Pearson’s rank correlation coefficient (r) and the corresponding significance level (*p*-value). The dietary exposure index for the study population was generated using a slightly different approach established by Shen et al. (2015) [61].

## 3. Results

### 3.1. Method Validation

The efficiency of the analytical method was investigated. Spiked and standard curves were constructed by using the ratio of the area of each compound and the area of internal standard. The system was linear for both spiked and standards solutions (R^2^ > 0.98) for all the analytes. The limits of detection (LOD) and quantification (LOQ) were estimated by analyzing the lowest spiked level for all the endocrine disruptors of this study and defined as the peaks that gave a signal-to-noise ratio ≥ 3 for LOD and ≥10 for LOQ. The LOD values ranged from 0.8 to 16.1 pg/mg for the PM, 3.1 pg/mg for TCS and 1.3 pg/mg for TCC. For PM, the LOQ values were ranged from 2.8 (MiBP) to 53.8 pg/mg (MEP), respectively, for TCS was 10.3 pg/mg and for TCC was 4.3 pg/mg (Table 1).

The validation of the applied method was performed at levels 25, 50, 100, 250, 500, 1000 and 2000 pg/mg and for TCS and TCC at levels 10, 25, 50, 100, 250, 500, 1000 for PM using blank head hair with no detected levels or levels close to the LOQ values of the method. Blank correction was performed by subtracting the signal obtained from the corresponding blank hair samples from the signal of the spiked hair samples prior to constructing the calibration curves. In addition, blank (solvent) samples and spiked samples were also included in each batch of samples to monitor potential background contamination and ensure the proper functioning of the instrument. The mean precision values (expressed as % relative standard deviation, %RSD) ranges from 11.6% (MCHP) to 18.8 (MiBP), the mean % recovery values ranged from 65.1 (MEP) to 124.3% (BTMHP) and the mean % accuracy values ranged from 86.5% (MCHP) to 118.4% (MEHP) (Table 1).

### 3.2. Demographics

A total of 212 children were enrolled in this study: control group (n = 51), T1D (n = 51), HPRD (n = 59), and OB (n = 51). The mean age of T1D children (11.2 ± 3.4 years) was significantly higher than that of the control group (9.4 ± 3.6 years). Children with T1D differed significantly in area of residence and maternal education compared with controls. A higher proportion of T1D children (58.8%) lived in rural areas than in controls and a higher proportion of T1D children had mothers with education up to the secondary-school level (62.7%) than in controls. The T1D children showed statistically significant increases in somatometric parameters, including WC, and in z-scores for height, weight, and BMI, compared with the control group. Lastly, SBP was higher in T1D children than in control-group children (Table 2).

The mean age of HPRD children was 10.1 ± 2.8 years and did not differ statistically from that of control children. The HPRD group of children differed significantly in area of residence (rural regions 52.5%) and in mother’s education compared to the control group (mothers with secondary education 71.2%). The HPRD children had statistically significantly higher WC and higher z-scores for waist-to-height ratio (WHR), height, weight and BMI than those in the control group. Moreover, the mean SBP concentration was significantly higher in the HPRD children (Table 2).

The mean age of OB children (10.6 ± 2.4 years) was significantly greater than that of children in the control group (9.4 ± 3.6 years). The proportion of mothers with secondary education was significantly higher in the OB group (64.7%) than in the control group (*p* < 0.01). In addition, the OB children had statistically significantly higher mean concentrations of WC and z-scores for height, weight and BMI. Likewise, the mean concentrations of WHR and SBP were significantly higher in the OB children than in the control group (Table 2).

### 3.3. Biomonitoring Data

The detection frequencies, the mean and median values of all analyzed EDCs for the positive samples are shown in Table S1 in Supplementary Materials.

Among studied EDCs, the MEHP, MBP, MiBP and TCS showed detection frequencies greater than 60% in the total population of children. In particular, the detection frequencies of MEHP, MBP, MiBP and TCS were 95.3%, 84.4%, 79.7%, and 60.8%, respectively. MEHP, MBP, MiBP and TCS levels were imputed with LOD/$\sqrt{2}$. Among total children’s hair, the mean concentrations of MEHP were 202.7 ± 311.4 pg/mg, of MBP were 52.3 ± 86.3 pg/mg, of MiBP in all children’s hair were 46.3 ± 60.5 pg/mg and of TCS were 231.7 ± 667.6 pg/mg.

Kruskall–Wallis results of all studies’ population group comparisons were *p* < 0.001 for MEHP and MiBP, *p* = 0.003 for MBP and *p* = 0.705 for TCS. Comparison of control groups to each disease group showed that the T1D and HPRD children provided higher mean MEHP concentration (217.5 ± 182.1 and 297.2 ± 476.7 pg/mg, respectively) than that of control children (175.4 ± 276.0 pg/mg), (*p* = 0.005 and 0.007, respectively). Statistically significant higher levels of MBP were also noticed for HPRD group compared to control group, while lower levels of MiBP were observed for OB (27.7 ± 25.9 pg/mg) compared to control group (49.1 ± 55.9 pg/mg) (*p* = 0.014) (Table 3).

### 3.4. Sex, Age, Area of Living and Maternal Education Comparison

The median concentrations of MEHP, MBP, MiBP and TCS were compared across discrete sociodemographic variables and disease groups, relative to the control group. Regarding the effect of children’s sex on PM concentrations, T1D boys had a higher median MEHP concentration (153.9 pg/mg) than control boys (97.6 pg/mg). The MBP levels in girls were higher in the HPRD group (51.5 pg/mg) and in T1D group (40.2 pg/mg) compared to the control. The OB boys had a lower median MiBP concentration (21.9 pg/mg) than the control boys (24.3 pg/mg).

A statistically significant increase in the median concentrations of MEHP and MBP was observed among HPRD children aged 2–10 years. In particular, the median concentrations of MEHP and MBP were 239.8 pg/mg and 56.9 pg/mg, respectively, which were higher than those of the 2–10-year-old control children (Table 4). No significant differences in median EDC concentrations were observed for the OB and T1D groups.

When the residential area was taken into consideration, urban T1D children showed a significant increase in the median MEHP concentration (153.1 pg/mg) compared with the control group. Likewise, urban HPRD children showed higher median concentrations of MEHP (237.6 pg/mg) and MBP (45.6 pg/mg) than the control group. The OB children showed lower median concentrations of MiBP (24.6 pg/mg) compared with those of the control group when children lived in rural regions (Table 4).

With respect to mothers’ education status, T1D children had higher median concentrations of MEHP (154.1 pg/mg) and MBP (47.0 pg/mg) than the control group when their mothers had a secondary education. The HPRD children showed higher median concentrations of MEHP (156.8 pg/mg), MBP (44.7 pg/mg) and TCS (94.1 pg/mg) than the control group while the OB children showed higher median concentrations of MBP (28.5 pg/mg) than those of control group (Table 4).

### 3.5. Somatometric and Clinical Characteristic Comparison

A significant negative correlation between MEHP and the BMI z-score (r = −0.37) was observed in control children; TCS also showed a negative association with the DBP z-score (r =−0.33) in control children. In the T1D group, MiBP levels were positively associated with normalized children’s height z-score (r = 0.41). Among the OB children, TCS levels were negatively correlated with z-scores for weight (r = −0.36), BMI (r = −0.31) and WHR (r = −0.34). In the HPRD group, age showed negative correlation with MEHP (r = −0.50), MBP (r = −0.38) and MiBP (r = −0.27). Application of Bonferroni correction showed significant *p*-values only in MEHP with children’ age in the HPRD group (Table 5).

The estimated cosmetics exposure index (CEI) was significantly and positively correlated with TCS in the control children (*p* = 0.025) and in OB children (*p* = 0.046). Regarding the relationship between PM levels and cosmetics use, CEI was significantly and inversely correlated with MBP levels in HPRD children (*p* = 0.011). Furthermore, no significant correlations between CEI and the examined biomarkers were observed in children with T1D (Table 6). When Bonferroni correction was applied, significant correlation for the HPRD–MBP pair remained (*p* = 0.045).

Correlation analyses revealed only a limited number of statistically significant associations between dietary group indices and hair PM levels across the study groups (Table 7). In the control group, consumption of starchy foods was positively correlated with MiBP levels (r = 0.31, *p* < 0.05). Processed-food consumption was positively correlated with MBP levels (r = 0.39, *p* < 0.05) in the T1D group and MEHP levels ((r = 0.31, *p* < 0.05) in the HPRD group. In OB children, processed food and sweets consumption was inversely correlated with MBP levels (r = −0.32, *p* < 0.05; r = −0.35, *p* < 0.05, respectively), while fruit and vegetables were inversely associated with MEHP levels (r = −0.30, *p* < 0.05). No significant correlations were observed after applying a Bonferroni correction.

## 4. Discussion

### 4.1. Hair Biomonitoring of PM, TCS and TCC During Childhood

We use hair biomonitoring to identify longer-term exposure to phthalates, TCC and TCS in children. In particular, we proceed to quantify PMs, TCS, and TCC in hair samples from Greek children aged 2 to 16 years with OB, T1D and HPRD. The novelty of this work lies in the simultaneous, multi-analyte evaluation of PMs, TCS and TCC across several different pediatric endocrine and metabolic diagnostic groups alongside a healthy control group. This comparative design offers preliminary evidence on EDC levels observed across different clinical groups of children. In contrast, most previous studies have compiled evidence on the distribution of PM, TCS, and TCC in children’s urine [62,63,64,65,66], recognizing that the urine matrix can show short-term EDC exposure [67,68,69]. As a result, this study quantifies EDCs during childhood via hair biomonitoring, as hair is an invaluable matrix that provides insights into longer-term EDC exposure, offering an assessment of EDC exposure over time [56,69,70,71,72,73,74,75,76].

In the total sample of 212 children, PMs were found in the following decreasing order of detection frequencies: MEHP > MBP > MiBP > MMP > BTMHP > MOP >MBzP > MCHP > MEP, implying widespread exposure to PMs during childhood. Among the investigated EDCs, detection frequencies exceeded 60% for MEHP, MBP, MiBP and TCS. TCC was rarely detected (3.8%), limiting its usefulness for group comparisons in this dataset. The main PM in children’s hair was MEHP, with a detection frequency of 95.3%, given that MEHP is the primary metabolite of di(2-ethylhexyl) phthalate (DEHP), which has endocrine-disrupting properties affecting development and reproduction [77]. Likewise, high MEHP values have been reported in other hair biomonitoring studies [78,79,80]. Following this, MBP and MiBP were detected at detection frequencies of 84.4% and 79.7% in children’s hair, respectively. As a result, the predominance of MEHP, MBP and MiBP is consistent with the prevalence of PMs in children, as reported in urine-based biomonitoring studies [35,64,66,81,82,83,84]. Overall, our findings provide evidence of mixed exposures to PM and TCS through hair biomonitoring, highlighting their prevalence during childhood.

### 4.2. The Levels of PM and TCS in OB Children

EDCs have been described as potential environmental “obesogens” and have drawn increasing attention as factors contributing to metabolic disturbance and the increased incidence of metabolic diseases [85].

Regarding the association between phthalate exposure and obesity, Grün and Blumberg introduced the concept of obesity-related effects in the action of EDCs [86]. A growing body of epidemiological studies has shown that obesity is consistently associated with phthalate exposure [83,87,88,89,90,91]. Several meta-analyses have reported associations between phthalate exposure and childhood adiposity [44,92]. On the one hand, prenatal phthalate exposure is associated with markers of OB and overweight later in childhood [93,94,95,96], whereas on the other hand, exposure to phthalates in children after birth is associated with insulin resistance, triglyceride accumulation, or hypertension [44,95,97,98,99,100,101,102,103,104,105]. Although a significant relationship between children’s exposure to phthalates and obesity has been reported, the direction and magnitude of the effects can vary by age, gender, living area, and PM class [91,102,106].

Among OB children enrolled in our study, our findings showed that OB children had a statistically significantly lower mean MiBP concentration than the control group, and this reduction was statistically significant among obese boys. In line with this, previous epidemiological studies have shown gender-specific associations between phthalate exposure and metabolic disorders in children, reinforcing the idea that phthalate exposure and metabolic changes in children are gender-dependent [88,102,107,108]. In a cohort study of Chinese children, the relationship between fat distribution and phthalate exposure appeared to be influenced by age and gender [107]. The urinary MBP levels appeared higher in boys, especially those aged 11–13 [107]. As a result, the above-mentioned urine biomonitoring study supported that boys’ adiposity was positively correlated with MBP concentrations in a concentration-effect manner, whereas girls’ adiposity was inversely correlated with MEHP concentrations [107]. Then, Buckley et al. showed that the effect of maternal urinary PM concentrations on adiposity was inverse only in girls [108]. Later, in another study conducted in Crete, sex-specific correlations between early life phthalate exposure and obesity were observed in children aged 4 years, with higher urine PM concentrations associated with lower BMI z-scores in boys and higher BMI z-scores in girls, implying that early life phthalate exposure is associated with changes in metabolism during childhood, depending on gender [109]. Recently, Dong et al. showed that children exposed to high concentrations of phthalates were more susceptible to becoming overweight, highlighting that this relationship was more consistent in females, indicating that increased phthalate exposure has been associated with metabolic disorders in a gender-specific manner [84]. In our study, no associations between PM levels and adiposity measures, such as z-scores for BMI and WC, were observed. Similar findings have been reported in Japanese children at the age of 7.5 years, in which previous exposure to PM mixtures when they were 1.5 and 3 years old did not substantially correlate with markers of adiposity, as evidenced by urine biomonitoring [110].

In addition, TCS levels appeared to be negatively associated with obesity-related somatometric parameters, including z-scores for weight, BMI, and WHR in obese children. In line with our findings, urinary TCS concentrations have inversely correlated with waist circumference and BMI in NHANES 2003–2010 [111]. In addition, fat mass percentage in 3-year-old children was inversely correlated with prenatal TCS exposure, as evidenced by urine biomonitoring [112]. However, null [113] or positive correlations between TCS exposure and adiposity during childhood have been reported in other studies [114,115].

### 4.3. The Levels of PM and TCS in Children with T1D

PMs have been implicated in metabolic and endocrinological pathways relevant to T1D [116]. According to the National Health and Nutrition Examination Survey (NHANES), specific urine PMs have been found to positively correlate with insulin resistance [117]. In recent decades, several epidemiological studies have established a relationship between higher urinary concentrations of specific PMs and new-onset or established T1D in children, probably through mechanisms involving oxidative stress in pancreatic β-cells, immune dysregulation, impaired insulin signaling, induction of adipogenesis, and endocrine disruption [116,118,119,120,121,122,123].

In our study, we found statistically significantly higher MEHP mean concentrations in children with T1D than in healthy children, with a mean increase of 1.24. Dufour et al. (2023) reported that increased levels of two DEHP metabolites, MEHHP and MEOHP, were positively correlated with glycated hemoglobin (HbA1c) and thyroid hormones in Belgian T1D children, providing evidence that phthalate metabolite exposure can be associated with impaired glycemic control and thyroid disturbance in this population [124].

Regarding the impact of living area on PM levels, our findings showed that MEHP levels were increased in T1D children, particularly those living in urban areas. Likewise, urine biomonitoring data have illustrated that healthy Danish children aged 6 to 11 years living in urban regions had higher levels of DEHP metabolite in urine [125]. As a result, our findings aligned with research indicating that T1D children living in urban areas may have increased exposure to phthalates, most likely due to differences in consumer products, food packaging, and lifestyles [125]. In addition, the median concentrations of MEHP and MBP were higher in T1D children whose mothers had completed secondary education. In line with this, an Irish study conducted on mother–child pairs showed that participant families with secondary education had considerably greater concentrations of PMs [126].

Urinary concentrations of PMs were reported to be negatively correlated with the height z-score in a gender-dependent manner, only among healthy Korean girls aged 7 to 14 [127]. Regarding the association between PM levels in T1D children and their somatotropic characteristics, MiBP levels in hair were positively correlated with z-scores for growth-related characteristics, such as height. This positive association between MiBP levels and the height of T1D children differed from that reported in another study, in which urinary levels of most PMs were inversely related to height in T1D children [128]. As a result, the inconsistent association between phthalates and growth-related characteristics can be explained by differences in biomonitoring matrices that reflect short-term or long-term EDC exposure.

Last but not least, the cardiovascular parameters are presented in relation to TCS exposure. In the T1D group, a negative association was observed between TCS levels and SBP z-score. While this may seem paradoxical, these results align with pediatric literature evaluating environmental chemical exposures. Specifically, Parastar et al. investigated cardiometabolic risk profiles in children and adolescents and reported a significant inverse relationship between dichlorophenol concentrations and pediatric SBP, given that 2,4-dichlorophenol is a TCS metabolite [129]. Furthermore, Nasab et al. have proposed a clear link between cardiovascular predictors in children and urinary 2,4-dichlorophenol, thereby substantiating the correlation between these antimicrobial contaminants and pediatric blood pressure [130]. For pediatric populations with chronic diseases, families emphasize high compliance with sanitation and health behaviors in clinical and metabolic management to stabilize their children’s cardiovascular factors [129,130]. In line with this, our findings highlight that PMs can inadvertently reflect behavioral habits rather than toxicological outcomes, underscoring the need for multivariate adjustments for lifestyle factors to delineate the potential impacts on children’s cardiovascular health.

### 4.4. The Levels of PM and TCS in Children with HPRD

Our hair biomonitoring data revealed increased mean concentrations of PMs, specifically MEHP and MBP, in HPRD children. In particular, children with hypothyroidism, growth hormone insufficiency, or adrenarche in urban areas had considerably higher MEHP and MBP levels.

Su et al. showed that urinary phthalate metabolite levels in eight-year-old children were tightly linked to serum progesterone levels, given that progesterone is involved in steroidogenesis as a precursor of testosterone [131]. In another study, increased detected levels of PM including MEHP and MBP were observed in children with constitutional delay of growth and puberty (CDGP) [132]. In these children, serum testosterone levels were substantially inversely correlated with urine concentrations of MBP and total PM [132].

In addition, our results showed that the median concentrations of selected PMs, including MEHP, MBP and MiBP, were statistically significantly and inversely associated with the age of children with HPRD. According to earlier research, urinary PM concentrations varied across age groups during childhood and were influenced by children’s behavior [64]. In addition, children’s dietary habits and use of personal-care products seemed to play a crucial role in regulating PM levels [133]. Last but not least, children’s MEHP, MBP and TCS median concentrations were associated with mother’s education, aligning with results from another Irish study showing that higher levels of children’s PM were linked to maternal secondary education [126].

### 4.5. The Exposure Sources of PMs and TCS Through Cosmetics Use and Dietary Habits

Children experience multiple concurrent phthalate exposures through diet and personal-care products, reinforcing the value of considering EDC exposure as a mixture rather than examining a single EDC compound [134,135]. In general, cosmetics and personal care items are recognized sources of exposure to TCS [136]. Our data showed a positive relationship between cosmetics use and hair TCS levels in the control and OB children. Our findings aligned with the knowledge that personal care products such as body washes, soaps and deodorants contain levels of TCS [76,137].

The current study demonstrated complex, group-specific differences between dietary habits and phthalate exposure across disease groups in children. Control children showed positive relationships between MiBP and starchy foods. Similar findings were reported in other studies, supporting the notion that childhood phthalate exposure is determined by the lipophilic nature of dietary fats that are delivered from paperboard packaging into humans [138,139]. Our findings also supported the role of processed foods as a source of EDCs in children with endocrine problems [90,140]. Finally, the OB children showed a negative correlation between the frequency of fruit and vegetable intake and MEHP concentration. Consistent with this, another epidemiological study by Rudel et al. showed that children who changed their regular dietary pattern to an intervention with fresh, unpackaged foods had a 53% drop in the urinary concentration of phthalic acid MEHP [141]. However, our findings showed negative correlations between processed foods and sweets and MBP. Despite these findings seeming paradoxical, they can reflect parental intervention in children’s dietary habits [142].

### 4.6. Limitations

Firstly, the hair TCS and TCC levels can be complicated, as the hair matrix can be affected by environmental contamination. External contamination, cosmetic treatments, and washing habits can impact hair TCS and TCC levels. To remove any external contamination, specific washing protocols are included in this hair biomonitoring study. Secondly, the cross-sectional design of this study limits the ability to establish a cause-and-effect relationship between EDCs and disease pathophysiology. Thirdly, the accuracy of the information on product use and dietary habits obtained from questionnaires completed by the children or their parents is difficult to verify.

## 5. Conclusions

Overall, our findings demonstrated differences in PM concentrations among Greek children across clinical groups of children through hair biomonitoring. In particular, MEHP, MBP, MiBP and TCS were the most frequently detected EDCs. Children with T1D and HPRD showed higher levels of specific PMs, especially MEHP and MBP, whereas OB children showed lower levels of MiBP. Our findings emphasized that PM concentrations differed among clinical groups and varied by gender, age, living area, maternal education, and the specific PM metabolite examined. Although OB children showed inverse relationships between TCS concentrations and somatometric parameters, TCS concentrations did not significantly differ between disease groups and controls. Regarding cosmetics exposure, OB children had higher TCS levels when they used cosmetics more frequently. Results differed across disease groups when accounting for diet. On the one hand, children with HPRD had increased MEHP levels while children with T1D had greater MBP levels when they consumed more processed foods. The OB group showed negative associations between processed foods/sweets and MBP levels. This biomonitoring study demonstrates distinct, group-specific EDC concentration patterns in pediatric hair samples. Given the cross-sectional nature of the study design, these associations do not establish causality or temporal directionality. Undoubtedly, further studies are needed to elucidate whether EDC concentrations can precede disease development or are related to disease-specific characteristics.

## Figures and Tables

**Table 1:** Analytical validation parameters of the applied methodology of PM, TCS and TCC.

Analytes	Rt (min)	Target *m*/*z*	QQ1 *m*/*z*	Standard Curves r^2^	Spiked Curves r^2^	Mean Recovery % (n = 3)	LOD (pg/mg)	LOQ (pg/mg)	Accuracy % (n = 4)	Inter-Day Precision (%RSD) (n = 4)
TCS	23.20	287.0	333.0	0.9996	0.9970	104.5	3.1	10.3	107.4	17.9
TCC	23.03	361.0	315.0	0.9992	0.9999	106.2	1.3	4.3	96.5	15.8
MMP	9.45	179.0	225.1	0.9861	0.9958	71.1	2.9	9.6	100.0	18.7
MEP	12.90	193.0	239.1	0.9806	0.9920	65.1	16.1	53.8	100.4	14.8
MiBP	17.10	221.1	267.1	0.9923	0.9919	101.0	0.8	2.8	99.9	18.8
MBP	17.26	221.1	267.1	0.9953	0.9948	99.9	1.3	4.2	109.5	17.6
MBzP	17.56	255.1	301.1	0.9887	0.9884	105.8	2.8	9.2	112.4	16.1
MCHP	18.45	247.1	293.1	0.9800	0.9951	116.3	4.5	15.0	86.5	11.6
MEHP	22.26	277.1	323.1	0.9961	0.9966	122.0	2.1	6.9	118.4	16.6
MOP	22.81	277.1	323.1	0.9892	0.9953	99.0	1.5	4.9	113.9	13.2
BTMHP	22.83	291.1	337.2	0.9915	0.9951	124.3	14.2	47.3	98.8	14.7
IS	14.15	231.1	-	-	-	-	-	-	-	-

Rt: retention time.

**Table 2:** Demographic and somatometric data for the disease group of children (T1D, HPRD, OB) compared to control group.

Variable		Control (n = 51)	T1D (n = 51)	HPRD (n = 59)	OB (n = 51)
		n (%)	n (%)	n (%)	n (%)
Child gender	Male	18 (35.3)	13 (25.5)	16 (27.1)	20 (39.2)
	Female	33 (64.7)	38 (74.5)	43 (72.9)	31 (60.8)
Area of living	Rural	14 (27.5)	30 (58.8) **	31 (52.5) **	20 (39.2)
	Urban	37 (72.5)	21 (41.2)	28 (47.5)	31 (60.8)
Mother’s education	Till secondary	18 (36.0)	32 (62.7) **	42 (71.2) **	33 (64.7) **
	University	32 (64.0)	19 (37.3)	17 (28.8)	18 (35.3)
		mean (SD)	mean (SD)	mean (SD)	mean (SD)
Child’s age	years	9.4 (3.6)	11.2 (3.4) *	10.1 (2.8)	10.6 (2.4) *
Child’s WC	cm	60.5 (9.6)	68.7 (10.1) **	71.5 (13.5) **	87.8 (11.2) **
WHR		0.5 (0.1)	0.5 (0.1)	0.5 (0.1) **	0.6 (0.1) **
Child’s height	z-score	−0.2 (1.3)	0.6 (1.0) **	0.1 (1.4) **	0.0 (1.4)
Child’s weight	z-score	0.0 (0.9)	0.9 (0.7) **	0.7 (1.5) **	0.7 (1.5) **
Child’s BMI	z-score	0.2 (1.0)	0.8 (0.8) **	0.9 (1.1) **	0.9 (1.2) **
SBP	z-score	−0.1 (1.1)	0.6 (1.0) **	1.0 (0.9) **	0.9 (1.0) **
DBP	z-score	0.3 (0.7)	0.7 (0.9)	0.5 (0.9)	0.5 (0.9)

WC: waist circumference; WHR: waist-to-hip ratio; BMI: body mass index; SBP: systolic blood pressure; DBP: diastolic blood pressure; z-score: standard score; * *p* < 0.05; ** *p* < 0.01.

**Table 3:** Detection frequencies, mean and median values (pg/mg) of PMs and TCS for each examined population group.

	Group	N (% Positive)	Mean ± SD (pg/mg)	Median (IQR) (pg/mg)	*p*	p*
MEHP	Total	202 (95.3)	202.7 ± 311.4	110.1 (55.8–230.7)		
	Control	47 (92.2)	175.4 ± 276.0	96.7 (47.7–173.1)		
	T1D	50 (98.0)	217.5 ± 182.1	161.1 (98.9–298.0)	0.005	0.517
	HPRD	58 (98.3)	297.2 ± 476.7	157.5 (84.9–276.6)	0.007	0.024
	OB	47 (92.2)	105.7 ± 123.1	60.9 (42.8–106.8)	0.095	0.147
MBP	Total	179 (84.4)	52.3 ± 86.3	32.4 (19.2–64.9)		
	Control	38 (74.5)	35.4 ± 34.8	28.7 (1.6–45.9)		
	T1D	33 (64.7)	48.2 ± 46.2	42.7 (1.6–79.0)	0.221	0.256
	HPRD	58 (98.3)	79.9 ± 146.3	44.7 (29.52–81.8)	0.001	0.010
	OB	50 (98)	41.5 ± 43.6	25.0 (19.7–37.5)	0.778	0.264
MiBP	Total	169 (79.7)	46.3 ± 60.5	31.8 (16.7–54.8)		
	Control	44 (86.3)	49.1 ± 55.9	32.8 (20.4–54.4)		
	T1D	25 (49.0)	48.8 ± 72.0	0.8 (0.8–86.0)	0.144	0.738
	HPRD	57 (96.6)	58.0 ± 71.5	42.6 (30.1–63.3)	0.128	0.208
	OB	43 (84.3)	27.7 ± 25.9	24.6 (14.8–31.8	0.014	0.050
TCS	Total	129 (60.8)	231.7 ± 667.6	28.6 (1.6–158.4)		
	Control	36 (70.6)	139.5 ± 416.3	7.3 (1.6–87.7)		
	T1D	27 (52.9)	409.1 ± 1147.4	7.3 (1.6–277.2)	0.828	0.164
	HPRD	38 (59.9)	214.6 ± 341.7	81.8 (1.6–267.1)	0.502	0.369
	OB	28 (54.9)	166.5 ± 458.6	7.3 (1.6–114.0)	0.768	0.840

SD: standard deviation; IQR: interquartile range; * Significance assessed with the Mann–Whitney test, p* after adjustment with age, residence and mother’s education.

**Table 4:** Subgroup analyses of median levels (pg/mg) of PMs and TCS in T1D, HPRD, and OB groups compared with the control group, stratified by sex, age, area of residence and maternal education status.

		Control			T1D			HPRD			OB		
		1st	Median	3rd	1st	Median	3rd	1st	Median	3rd	1st	Median	3rd
	Gender												
Boys	MEHP	23.0	97.6	133.1	134.9	153.9 *	284.7	75.1	120.9	208,5	42.7	85.6	134.2
	MBP	18.6	33.5	47.4	31.5	79	103.7	27	32.4	54.1	17.8	22.5	33.6
	MiBP	24.3	31.8	43.3	0.8	40.3	78.7	27.2	36.4	60.2	16.2	21.9 *	29.6
	TCS	1.6	7.3	48.9	1.6	1.6	231.7	1.6	72.8	229.3	1.6	1.6	18.2
Girls	MEHP	50.2	95.1	240.5	98.6	163.5	298	84.9	178.9	300	43.7	60.9	104.2
	MBP	1.6	26	42.1	1.6	40.2 *	63.3	30.1	51.5 *	81.9	20.4	25.3	53.8
	MiBP	19.8	34.7	54.4	0.8	0.8	86	30.1	44.2	68.5	15.8	26.6	38.4
	TCS	1.6	20.3	104.4	1.6	44.5	277.2	1.6	89	267.1	1.6	42.5	215.8
	Age												
2–10	MEHP	55.6	98.4	157.1	84.4	150.1	217.0	132.1	239.8 *	448.2	45.5	80.5	145.6
	MBP	18.6	33.0	45.9	1.6	35.7	80.8	34.8	56.9 *	91.7	18.1	22.1	53.8
	MiBP	20.4	32.8	54.4	0.8	0.8	58.6	33.2	45.1	83.2	9.3	22.7	29.5
	TCS	1.6	7.3	56.0	1.6	141.5	622.6	1.6	93.2	280.4	1.6	24.1	144.6
11–16	MEHP	47.7	92.1	177.0	100.8	177.7	370.9	69.1	100.8	178.2	42.5	57.1	105.4
	MBP	1.6	22.5	53.4	1.6	44.9	79.0	21.8	33.9	61.0	20.9	26.4	38.7
	MiBP	21.1	32.7	53.4	85.3	0.8	98.8	24.3	36.3	54.5	17.9	27.5	37.7
	TCS	1.6	7.3	120.0	1.6	1.6	231.7	1.6	68.7	152.9	1.6	20.9	102.8
	Living area												
Rural Urban	MEHP	47.7	92.9	363.7	131.9	184.4	298	53.9	123	182.9	52.5	81.4	138.1
	MBP	14.9	24	59.1	1.6	47.5	79	22	42.2	79.2	19.5	21.9	36.9
	MiBP	13.8	30.7	78.5	0.8	17.8	90.4	24.9	41.7	57.1	16.6	25.8	34.7
	TCS	1.6	7.3	128.5	1.6	18	265	1.6	90.9	267.1	1.6	26.3	115.2
	MEHP	50.2	96.7	142.1	91	153.1 *	284.7	98.1	237.6 *	324.6	42.3	49.7	106.6
	MBP	1.6	30.7	38.8	1.6	31.5	62.9	31	45.6 *	86.4	20.3	25.3	53.8
	MiBP	22.4	34.7	51.4	0.8	0.8	55	32	44.5	65.9	15.8	24.6 *	31.8
	TCS	1.6	7.3	57.5	1.6	7.3	277.2	1.6	73.1	276	1.6	7.3	114
	Mother’s education												
Till Secondary	MEHP	39.2	76.5	142.1	99.9	154.1 *	227.1	85.2	156.8 *	273.9	45.5	82.2	145.6
	MBP	1.6	21.8	33.9	1.6	47.0 *	101	30.3	44.7 *	81.8	20.3	28.5 *	53.8
	MiBP	19.5	31.3	42.3	0.8	0.8	84.6	33	44.3	59.2	14.4	24.2	35.1
	TCS	1.6	1.6	30.7	1.6	18	517.1	1.6	94.1 *	277.9	1.6	7.3	91.6
University	MEHP	62.9	102.4	175.1	91	177.9	325.8	69.1	178.9	283.8	38.7	48.4	88.9
	MBP	18.3	32.4	53.9	1.6	29.3	75.5	19.1	41.3	67.2	19.9	22.5	30.6
	MiBP	20/8	34	66.6	0.8	29.7	86	22.2	31.8	63.3	20	26.6	29.9
	TCS	7.3	26.3	82.1	1.6	7.3	177.6	1,6	64.4	109.1	1.6	33.1	138.7

* *p* < 0.05, 1st and 3rd quartile.

**Table 5:** Correlation of MEHP, MBP, MiBP and TCS levels with children’s age and normalized somatometrics, in T1D, HPRD, and OB and control children.

		MEHP (r)	MBP (r)	MiBP (r)	TCS (r)
Control	Age (years)	−0.12	−0.20	−0.13	0.09
	Height ^#^	−0.05	0.00	−0.09	0.01
	Weight ^#^	−0.17	−0.06	−0.21	0.04
	BMI ^#^	−0.37 *	−0.17	−0.27	0.08
	WHR	0.09	0.26	0.06	0.00
	SBP ^#^	−0.20	−0.14	−0.13	−0.20
	DBP ^#^	−0.07	0.10	−0.02	−0.33 *
T1D	Age (years)	0.18	0.12	0.05	−0.13
	Height ^#^	0.04	0.00	0.41 *	0.09
	Weight ^#^	0.06	0.07	0.10	0.13
	BMI ^#^	0.01	0.15	−0.14	0.11
	WHR	0.02	−0.21	−0.12	0.15
	SBP ^#^	0.06	0.25	0.26	−0.14
	DBP ^#^	0.06	0.14	0.06	−0.10
HPRD	Age (years)	−0.50 *^,^**	−0.38 *	−0.27 *	−0.11
	Height ^#^	−0.08	−0.01	−0.04	0.02
	Weight ^#^	−0.07	−0.04	−0.11	−0.09
	BMI ^#^	−0.03	−0.06	−0.12	−0.11
	WHR	0.05	0.08	0.04	−0.07
	SBP ^#^	0.09	−0.01	−0.07	−0.11
	DBP ^#^	−0.02	−0.09	0.02	0.02
OB	Age (years)	−0.16	−0.04	0.12	0.01
	Height ^#^	−0.06	−0.08	−0.16	−0.22
	Weight ^#^	0.14	−0.05	−0.24	−0.36 *
	BMI ^#^	0.25	0.03	−0.25	−0.31 *
	WHR	0.26	−0.01	−0.08	−0.34 *
	SBP ^#^	0.05	0.07	−0.14	−0.12
	DBP ^#^	0.08	−0.04	−0.19	−0.06

* *p* < 0.05, ^#^ z-score, r = Spearman’s rank correlation coefficient ** *p* < 0.05 after Bonferroni correction.

**Table 6:** Correlation of MiBP, MBP, MEHP and TCS levels in investigated groups compared to those of control group with cosmetics exposure index (CEI).

Group	Control		T1D		HPRD		OB	
	r	*p*	R	*p*	r	*p*	r	*p*
MiBP	0.011	0.942	0.026	0.859	−0.167	0.205	0.008	0.958
MBP	−0.086	0.551	−0.033	0.817	−0.327	0.011 (0.045)	−0.069	0.630
MEHP	0.024	0.868	−0.102	0.476	−0.098	0.459	−0.001	0.996
TCS	0.315	0.025	0.118	0.411	0.218	0.097	0.281	0.046

CEI: cosmetics exposure index; r: Spearman’s rank correlation coefficient. (): Bonferroni corrected *p* values.

**Table 7:** Correlation coefficients between food-group-consumption indices and hair PM concentrations, stratified by study group.

Population Group		Food Group DEI										
		Dairy	Meat	Fish	Starchy Foods	Processed Foods	Fruits/ Vegetables	Legumes	TFC	Sweets	Refreshments	Eggs
Control	MEHP	−0.06	0.03	−0.08	0.11	0.17	−0.07	−0.08	0.13	0.02	0.15	−0.17
	MBP	0.12	0.21	0.16	0.09	−0.03	−0.03	0.17	0.18	0.03	0.09	−0.15
	MiBP	0.02	0.15	0.17	0.31 *	0.05	−0.21	0.21	0.08	−0.01	0.04	−0.25
T1D	MEHP	−0.05	0.00	−0.08	−0.18	0.17	−0.11	−0.14	−0.17	−0.14	−0.12	0.24
	MBP	0.15	0.05	0.00	0.09	0.39 *	−0.05	0.01	0.24	0.06	0.15	0.17
	MiBP	0.13	0.25	0.07	0.11	0.22	−0.14	0.10	0.03	0.08	0.16	−0.05
HPRD	MEHP	−0.14	−0.06	−0.20	−0.17	0.31 *	0.02	0.17	0.08	−0.11	0.12	−0.09
	MBP	0.13	−0.04	−0.08	0.11	0.16	−0.05	−0.01	−0.01	−0.03	0.02	−0.04
	MiBP	0.13	−0.02	−0.14	0.17	0.14	−0.12	0.15	0.07	0.06	−0.03	−0.07
OB	MEHP	0.07	−0.08	−0.23	0.00	0.18	−0.30 *	0.11	−0.13	0.06	−0.01	0.05
	MBP	0.01	−0.25	−0.10	−0.10	−0.32 *	−0.19	0.10	−0.22	−0.35 *	−0.05	−0.06
	MiBP	−0.01	0.14	−0.18	−0.02	−0.02	−0.11	0.21	0.06	0.15	−0.07	0.17

* *p* < 0.05.

## Data Availability

The original contributions presented in this study are included in the article. Further inquiries can be directed to the corresponding authors.

## Supplementary Materials

The following supporting information can be downloaded at https://www.mdpi.com/article/10.3390/jox16050158/s1, Table S1: PMs, TCS and TCC detection frequencies, mean and median values (pg/mg) of positive hair samples for T1D, HPRD and OB groups compared to control children.

## Abbreviations

AAP	American Academy of Pediatrics
APCI	Atmospheric pressure chemical ionization
BMI	Body mass index
BTMHP	Bis(3,5,5-trimethylhexyl) phthalate
CDC	Centers for Disease Control and Prevention
CEI	Cosmetics exposure index
DBP	Diastolic blood pressure
DEI	Dietary exposure index
DEHP	Di(2-ethylhexyl) phthalate
DNA	Deoxyribonucleic acid
DOHaD	Developmental origins of health and disease
EDCs	Endocrine-disrupting chemicals
FFQ	Food frequency questionnaire
HbA1c	Glycated hemoglobin
HPA	Hypothalamic–pituitary–adrenal axis
HPG	Hypothalamic–pituitary–gonadal axis
HPRD	Hypothalamus–pituitary–related disorders
HPS	Hypothalamic–pituitary–somatotropic axis
HPT	Hypothalamic–pituitary–thyroid axis
IBM	International Business Machines
IQR	Interquartile range
LC-MS	Liquid chromatography-mass spectrometry
LOD	Limit of detection
LOQ	Limit of quantification
MBP	Monobutyl phthalate
MBzP	Monobenzyl phthalate
MCHP	Monocyclohexyl phthalate
MEHHP	Mono(2-ethyl-5-hydroxyhexyl) phthalate
MEHP	Mono(2-ethylhexyl) phthalate
MEOHP	Mono(2-ethyl-5-oxohexyl) phthalate
MEP	Monoethyl phthalate
MiBP	Mono-isobutyl phthalate
MMP	Monomethyl phthalate
MOP	Mono-n-octyl phthalate
mmHG	Millimeters of mercury
NIS	Sodium iodide symporter
OB	Obesity
*p*-value	Corresponding significance level
PMs	Phthalate metabolites
%RSD	Percent relative standard deviation
RT	Retention time
SBP	Systolic blood pressure
SD	Standard deviation
SIM	Selected ion monitoring
SPSS	Statistical Package for the Social Sciences
T1D	Type 1 diabetes mellitus
T4	Thyroxine
TCC	Triclocarban
TCS	Triclosan
TFC	Fat and condiments
TPO	Thyroid peroxidase
TRC	Toronto Research Chemicals
TRH	Thyrotropin-releasing hormone
TSH	Thyroid-stimulating hormone
WC	Waist circumference
WHR	Waist-to-hip-ratio
z-score	Standardized SD score
