# Supplementary material for: Hair-Based Biomonitoring of Phthalate Metabolites and Triclosan in Childhood: Links with Metabolic and Endocrine Disorders
Source: J Xenobiot. 2026 Aug 25;16(5):158. doi: 10.3390/jox16050158 (PMC13608689; doi:10.3390/jox16050158)
Supplement: Supplementary file 1 [file jox-16-00158-s001.zip › jox-4502565-supplementary.pdf]

Hair-Based Biomonitoring of Phthalate Metabolites and Triclosan in Childhood: Links with Metabolic and Endocrine Disorders

Michail Koukakis, Stella Baliou, Athanasios Alegakis, Elena Vakonaki, Dimitra Volakaki, Ilianna Maniadaki, Eleftheria Papadopoulou, Dimitris Mamoulakis, Marilia Lioudaki, Emmanouil Paraskakis, Ioannis Germanakis, Aristides Tsatsakis and Manolis N. Tzatzarakis

Table S1. PMs, TCS and TCC detection frequencies, mean and median values (pg/mg) of positive hair samples for T1D, HPRD and OB groups compared to control children.

	Group	N (% positive)	Mean ± SD (pg/mg)	Median (IQR) (pg/mg)	p-value
MEHP	Total	202 (95.3)	212.6 ± 315.7	122.9 (68.5-238.7)	
	Control	47 (92.2)	190.2 ± 282.7	101.4 (57.3-177.0)	
	T1D	50 (98.0)	221.8 ± 181.3	163.5 (100.8-298.0)	0.015
	HPRD	58 (98.3)	302.3 ± 479.2	159.1 (85.2-276.6)	0.022
	OB	47 (92.2)	114.6 ± 124.3	66.3 (44.0-122.8)	0.068
MBP	Total	179 (84.4)	61.7 ± 90.9	38.8 (23.8-78.2)	
	Control	38 (74.5)	47.0 ± 33.1	35.5 (26.0-59.1)	
	T1D	33 (64.7)	73.7 ± 38.0	63.3 (44.9-103.7)	0.002
	HPRD	58 (98.3)	81.3 ± 147.3	45.1 (30.1-81.8)	<0.001
	OB	50 (98)	42.2 ± 43.6	25.1 (19.9-37.5)	0.067
MiBP	Total	169 (79.7)	57.9 ± 62.7	40.1 (26.6-63.6)	
	Control	44 (86.3)	56.7 ± 56.5	38.5 (26.7-63.0)	
	T1D	25 (49.0)	98.8 ± 75.5	86.0 (49-114.7)	<0.001
	HPRD	57 (96.6)	60.0 ± 71.9	43.2 (31.8-63.3)	0.469
	OB	43 (84.3)	32.7 ± 25.2	26.6 (20.0-35.1)	0.002
TCS	Total	129 (60.8)	379.8 ± 823.6	114.0 (43.4-393.0)	
	Control	36 (70.6)	196.9 ± 485.9	46.1 (7.3-124.3)	
	T1D	27 (52.9)	771.2 ± 1497.4	265.0 (86.2-721.0)	<0.001
	HPRD	38 (59.9)	332.2 ± 378.2	147.9 (89.0-425.4)	<0.001
	OB	28 (54.9)	301.9 ± 589.3	92.3 (31.8-354.5)	0.094
MMP	Total	46 (21.7)	77.3 ± 87.0	45.3 (22.4-82.9)	
	Control	19 (37.3)	48.2 ± 23.7	43.7 (28.0-68.9)	
	T1D	15 (29.4)	106.9 ± 114.1	60.6 (17.1-193.4)	0.560
	HPRD	8 (13.6)	85.2 ± 99.8	50.8 (12.2-123)	0.897
	OB	4 (7.8)	88.3 ± 127.3	29.2 (20.8-155.9)	0.456
BTMHP	Total	31 (14.6)	48.4 ± 56.1	15.6 (15.6-73.5)	
	Control	5 (9.8)	78.9 ± 112.0	15.6 (15.6-73.5)	
	T1D	16 (31.4)	42.8 ± 39.9	15.6 (15.6-74.0)	0.842
	HPRD	8 (13.6)	34 ± 26.4	15.6 (15.6-57.3)	0.724
	OB	2 (3.9)	75 ± 84.0	75 (15.6-134.4)	0.857
MOP	Total	25 (11.8)	120.9 ± 158.8	49.9 (21-185.9)	
	Control	9 (17.6)	123 ± 134.9	27.4 (20.4-207.6)	
	T1D	7 (13.7)	89.2 ± 73.2	54.1 (44.9-185.9)	0.681
	HPRD	4 (6.8)	254.8 ± 322.3	146 (22.7-486.9)	0.503
	OB	5 (9.8)	54.3 ± 61.2	14.2 (14.2-75.9)	0.298
MBzP	Total	19 (9.0)	38.9 ± 19.4	35.0 (21.7-51.2)	

	Group	N (% positive)	Mean ± SD (pg/mg)	Median (IQR) (pg/mg)	p-value
MBzP	Control	7 (13.7)	37 ± 13.3	35.7 (23.9-51.2)	
	T1D	4 (7.8)	63.7 ± 23.7	69.9 (47.1-80.4)	0.073
	HPRD	7 (11.9)	30.2 ± 8.5	33.8 (21.6-38.3)	0.383
	OB	1.0 (2.0)	14.2 ± 0.0	14.2 (14.2-14.2)	0.25
TCC	Total	8 (3.8)	17.2 ± 23.0	10.8 (3.1-17.2)	
TCC	Control	2 (3.9)	12.9 ± 7.6	12.9 (7.5-18.2)	
	T1D	0 (0.0)	NE	NE	NE
	HPRD	2 (3.4)	8.6 ± 7.8	8.6 (3.1-14.1)	0.667
	OB	4 (7.8)	23.6 ± 32.9	9.7 (3.1-44.1)	0.800
MCHP	Total	9 (4.2)	78.0 ± 40.0	76.8 (45.2-98.3)	
	Control	0 (0.0)	NE	NE	
	T1D	5 (9.8)	78.3 ± 29.3	91.1 (66.3-98.3)	NE
	HPRD	4 (6.8)	77.6 ± 55.8	61.0 (38.6-116.6)	NE
	OB	0 (0.0)	NE	NE	NE
MEP	Total	8 (3.8)	95.6 ± 99.1	67.7 (45.2 -77.9)	
	Control	1 (2.0)	66.7 ± NE	66.7 (66.7-66.7)	
	T1D	6 (11.8)	108.9 ± 113.4	69.5 (45.2 -85.6)	0.857
	HPRD	1 (1.7)	45.2 ± NE	45.2 (45.2-45.2)	1.000
	OB	0 (0.0)	NE	NE	0.606

SD: standard deviation; IQR: interquartile range; NE: not-estimated;* Significance assessed with the Mann-Whitney test.
